# How Common Is Disorder? Occurrence of Disordered Residues in Four Domains of Life

**DOI:** 10.3390/ijms160819490

**Published:** 2015-08-18

**Authors:** Mikhail Yu. Lobanov, Oxana V. Galzitskaya

**Affiliations:** Institute of Protein Research, Russian Academy of Sciences, Pushchino 142290, Moscow Region, Russia; E-Mail: mlobanov@phys.protres.ru

**Keywords:** proteome, homo-repeats, disordered regions, computational prediction

## Abstract

Disordered regions play important roles in protein adaptation to challenging environmental conditions. Flexible and disordered residues have the highest propensities to alter the protein packing. Therefore, identification of disordered/flexible regions is important for structural and functional analysis of proteins. We used the IsUnstruct program to predict the ordered or disordered status of residues in 122 proteomes, including 97 eukaryotic and 25 large bacterial proteomes larger than 2,500,000 residues. We found that bacterial and eukaryotic proteomes contain comparable fraction of disordered residues, which was 0.31 in the bacterial and 0.38 in the eukaryotic proteomes. Additional analysis of the total of 1540 bacterial proteomes of various sizes yielded a smaller fraction of disordered residues, which was only 0.26. Together, the results showed that the larger is the size of the proteome, the larger is the fraction of the disordered residues. A continuous dependence of the fraction of disordered residues on the size of the proteome is observed for four domains of life: Eukaryota, Bacteria, Archaea, and Viruses. Furthermore, our analysis of 122 proteomes showed that the fraction of disordered residues increased with increasing the length of homo-repeats for polar, charged, and small residues, and decreased for hydrophobic residues. The maximal fraction of disordered residues was obtained for proteins containing lysine and arginine homo-repeats. The minimal fraction was found in valine and leucine homo-repeats. For 15-residue long homo-repeats these values were 0.2 (for Val and Leu) and 0.7 (for Lys and Arg).

## 1. Introduction

Prediction of 3D protein structure and function is a general direction of structural genomics. Of special interest is the search for important functional regions and motifs in a polypeptide chain. To this end, several databases are currently available: PROSITE, InterPro, Pfam, and ModiDP [1,2,3,4]. Protein regions without a fixed structure, a.k.a. disordered regions, often play important functional roles, yet such regions cannot be resolved by X-ray crystallography. Therefore, prediction of disordered regions for various proteomes is necessary to identify fragments of globular proteins amenable to crystallization, as well as to understand protein functions and their environmental adaptability.

Protein adaptation to extreme conditions is achieved via the major functional simplification apparent at the level of both the genome and the individual genes and proteins [5,6]. The adaptation of the catalytic, structural and regulatory functions of proteins to extreme conditions (high or low temperatures, salinity, *etc.*) is of particular interest. To understand the molecular mechanism of the protein adaptation, one should either rely on the experimentally determined 3D structures of the proteins and/or analyze their sequences. Here we carried out sequence analyses of 122 eukaryotic and bacterial proteomes to predict their disordered regions.

We created the first library of disordered regions based on the known protein structures from the clustered protein data bank [7,8]. The library currently contains 171 disordered patterns. Most of such patterns comprise amino acid motifs with low complexity, including homo-repeats that are known to be disordered [8,9]. One example is poly-His repeats that may play important functional roles. For example, six consecutive histidines facilitate efficient purification of a recombinant protein on a nickel column [10]. Moreover, functional histidine repeats frequently occur in the human proteome [7]. The minimal length of the homo-repeats that can influence the protein structure and function is thought to be 5–7 residues [7,10,11]. Increasing the length of homo-repeats can lead to enhanced protein aggregation and amyloid fibril formation [12]. Therefore, the presence of long repeats in proteins is often linked to amyloid diseases. For example, Huntington’s disease [13] involves long poly-glutamine repeats in protein huntingtin, whereas ocular muscular dystrophy involves poly-alanine repeats in polyadenine-binding protein 2 [14,15].

Recently we analyzed the occurrence of the disordered patterns in 122 eukaryotic and bacterial proteomes to create the HRaP database [16]. Furthermore, we proposed a new phyloproteomic criterion based on the occurrence of simple motifs that can be imprints of evolutionary history. We studied the occurrences of 11,780 six-residue-long motifs consisting of two randomly located amino acids in 97 eukaryotic and 25 bacterial proteomes [17]. Here we address two questions. First, how many disordered residues are there in 122 eukaryotic and bacterial proteomes? Second, is it possible to change the dependence of the fraction of the disordered residues on the length of the protein homo-repeat?

## 2. Results and Discussion

### 2.1. How Common Is Disorder? Prediction of Disordered Residues in 122 Proteomes

Previously, we analyzed the occurrence of 171 various disordered patterns in 122 eukaryotic and bacterial proteomes. Among those, 23 are found only in the PDB but not in the actual proteomes, indicating that these patterns have been artificially added: WSHPQFEK, GMDELYK, SAWSHPQF, ASMTGGQQMGR, HHHHHHSQDP, HHHHHMA, TSLYKKAG, GGLNDIFEAQKIEWH, HHHHHHHHHSSGHIDDDDKHM, ENLYFQGS, EQKLISEEDLN, ENLYFQGHM, SHMAS, AMADIGS, GSHMLEDP, GEKHHHHH, HIEGRH, HHHHHHSSGLEVLFQGP, PTTENLYFQGAM, EGGHHHHH, HHHHHGGS, DCGCKPCI, and IKSHHNVGGLP. The patterns with the most frequent occurrence in the eukaryotic proteomes have low complexity: PPPPP, GGGGG, EEEED, HHHH, KKKKK, SSTSS, and QQQQQP.

**Figure 1:**
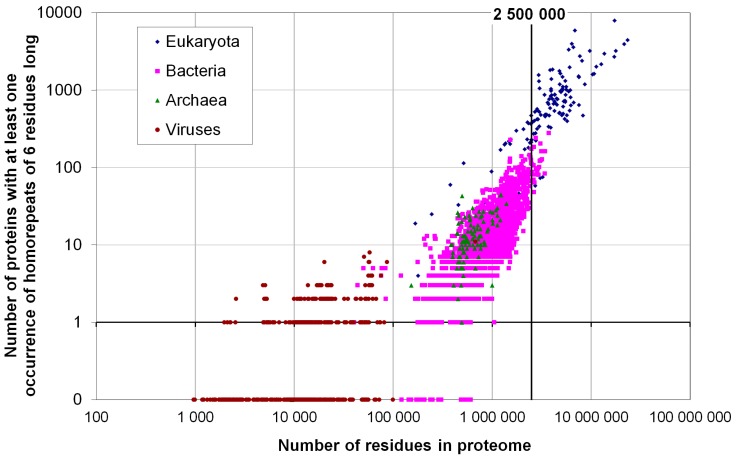
Number of proteins with at least one occurrence of homo-repeat of 6 residues long for 1902 proteomes from eukaryotes (137), bacteria (1540), archaea (105) and viruses (120).

We calculated the occurrence of homo-repeats of 6 residues or longer for 20 different amino acids. The results in Figure 1 reveal that such occurrence is more frequent in the eukaryotic than in the bacterial proteomes, and show a well-defined boundary between these proteomes.

**Figure 2:**
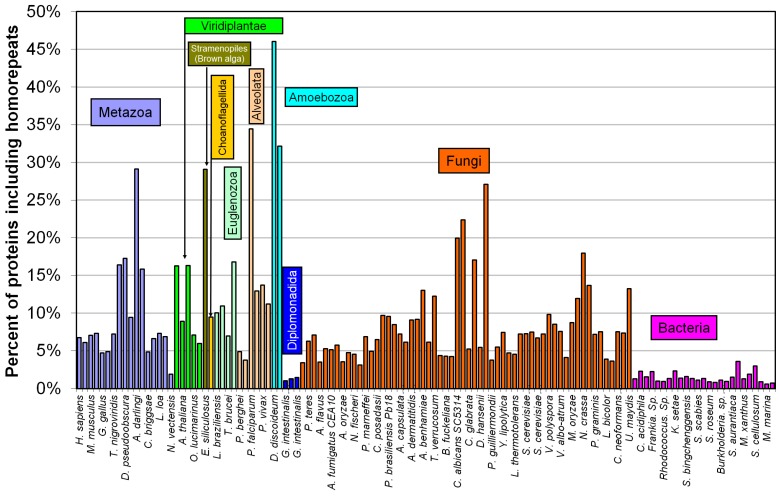
Percent of proteins including homo-repeats for 122 proteomes.

We also analyzed such occurrences in different kingdoms (see Figure 2).

**Figure 3:**
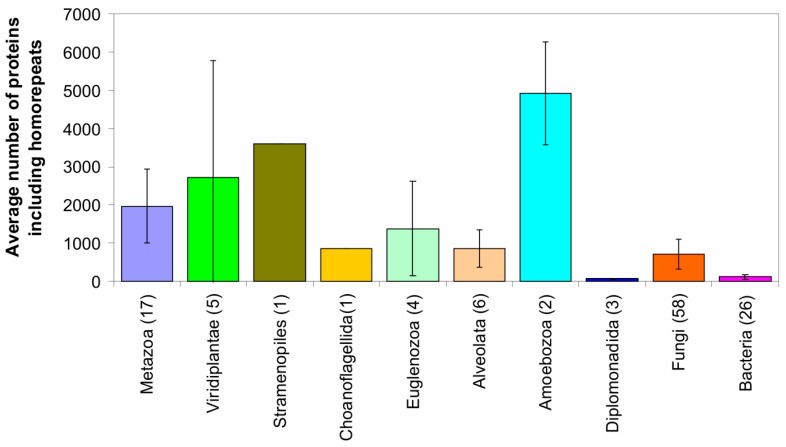
Occurrence of homo-repeats of 6 residues long for 20 amino acids in 122 proteomes. Numbers of proteomes are indicated in the parentheses. Error bars are standard deviations.

The leaders among the nine eukaryotic kingdoms and five bacterial taxonomic groups are amoebozoa and alveolata proteomes (Figure 2 and Figure 3). In amoebozoa proteomes, nearly one half of all proteins include homo-repeats of 6 residues or longer. In alveolata proteomes, one third of all proteins contain such homo-repeats.

**Figure 4:**
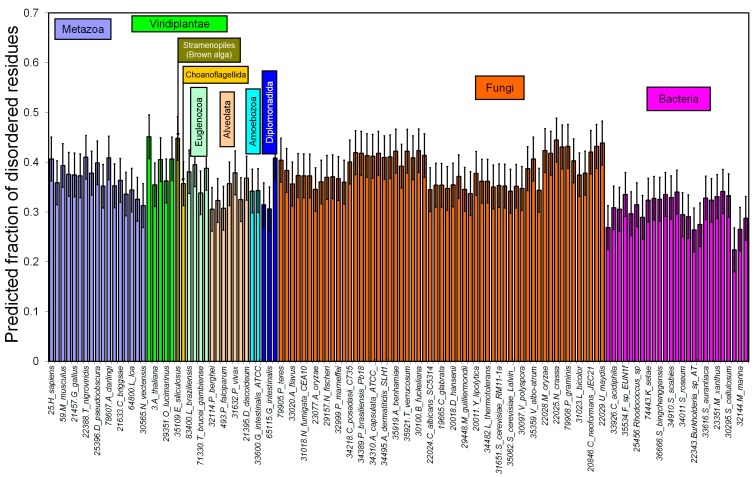
Predicted fraction of disordered residues for 122 proteomes. Error bars are standard deviations.

We predicted the disordered residues for 122 proteomes using the IsUnstruct program [18,19]. For each residue the probability to be unfolded was calculated. The sum of these probabilities normalized to the protein length gives the fraction of the disordered residues in a protein. Surprisingly, for the 25 bacterial proteomes analyzed, the fraction of disordered residues is also high, 0.31 on average (Figure 4). Perhaps such a large fraction of disordered residues is typical for large bacterial proteomes such as those analyzed in our work, with the total number of residues exceeding 2,500,000.

When the predictions were made by using the modified potentials which consider the effect of overprediction of disordered residues in the terminal regions, then similar results are obtained for all 122 proteomes. Only for bacterial proteomes fraction of disordered residues increased from 0.31 to 0.36. Therefore, such a large fraction of disordered residues identified in the bacterial proteomes is not a result of overprediction for the protein termini.

In the previous study we predicted the percent of proteins with disordered regions larger than 41 residues using the FoldUnfold program [20]. Based on our estimates, 12%, 3% and 2% of the proteins in eukaryotic, bacterial and archaean proteomes, respectively, are totally disordered. Long (over 41 residues) disordered segments were found in 16% of arhaean, 20% of bacterial, and 43% of eukaryotic proteins when using 19 archaean, 159 bacterial and 17 eukaryotic proteomes [21].

**Figure 5:**
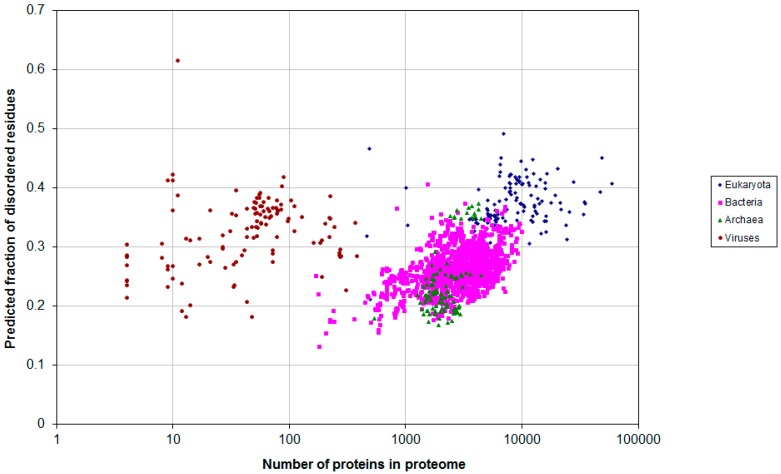
Predicted average fraction of disordered residues for 1902 species from eukaryotes (137), bacteria (1540), archaea (105) and viruses (120) *versus* the proteome size.

Next, we considered all proteomes available at the UniProt database. Upon increasing the number of bacterial proteomes from 25 to 1540, fraction of disordered residues decreased from 0.31 to 0.26. Moreover, upon increasing the number of eukaryotic proteomes from 97 to 137, this fraction also decreased from 0.38 to 0.36. This suggests that large bacterial proteomes have a fraction of disordered residues comparable to that in the eukaryotic proteomes (see Figure 5). For 105 archaean proteomes this fraction was 0.24, and for 120 viral proteomes it was 0.28. Notably, eukaryotic proteomes have large-scale variations in their proteome sizes, from 463 to 59,053 proteins, whereas the size of bacterial proteomes ranges from 182 to 10,019 proteins.

Similar analysis by using the PONDR-VSL2B program was reported in [22] where 3484 proteomes were considered. Eukaryotes were reported to have 32% disordered residues, whereas prokaryotes had 27%, suggesting that the boundary between the prokaryotic and eukaryotic proteomes was approximately at 30%. In our case, by using the IsUnstruct program to analyze 137 eukaryotic and 1540 bacterial proteomes, the predicted fraction of disordered residues was 0.36 and 0.26, respectively, suggesting a good agreement between [22] and our studies for the proteomes considered. However, for very large bacterial proteomes (>2,500,000 residues), the fraction of disordered residues was 0.31, comparable to that in eukaryotic proteomes reported in [22].

**Figure 6:**
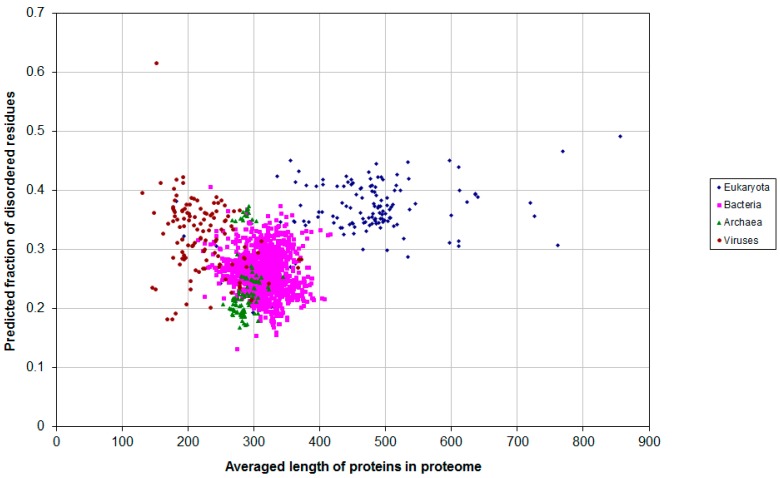
Predicted average fraction of disordered residues for 1902 species from eukaryotes (137), bacteria (1540), archaea (105) and viruses (120) *versus* the average protein length in the proteome.

Figure 6 shows the predicted average fraction of disordered residues plotted as a function of the average protein length in the proteome. The distributions for the eukaryotic, bacterial, archaean and viral proteomes overlap at about 0.3–0.4 fraction disordered, even though the average protein length is longer for eukaryotic proteomes (Figure 6).

**Figure 7:**
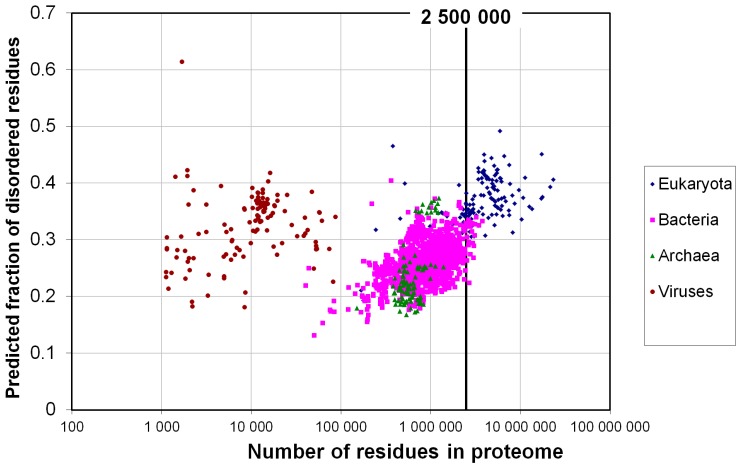
Predicted average fraction of disordered residues for 1902 species from eukaryotes (137), bacteria (1540), archaea (105) and viruses (120) *versus* the average protein length in the proteome.

Figure 5, Figure 6 and Figure 7 show that archebacteria are divided into two groups: halobacteria and others. Halobacterial proteomes have high fraction of disordered residues, which may reflect the adaptation to environmental conditions [23].

In contrast to a recent study [22], we did not observe a sharp increase in the fraction of disordered residues upon transition from prokaryotic to eukaryotic proteomes. Instead, Figure 7 shows continuous dependence of the fraction of disordered residues on the size of the proteome.

### 2.2. Fraction of Disordered Residues in Proteins Containing Homo-Repeats of Different Length

**Figure 8:**
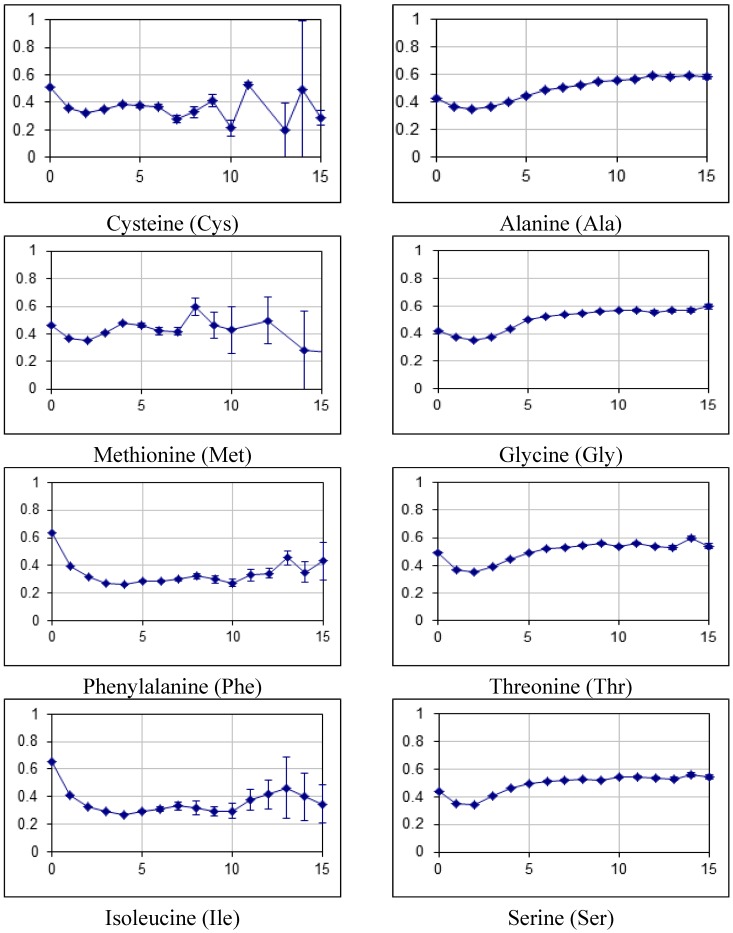

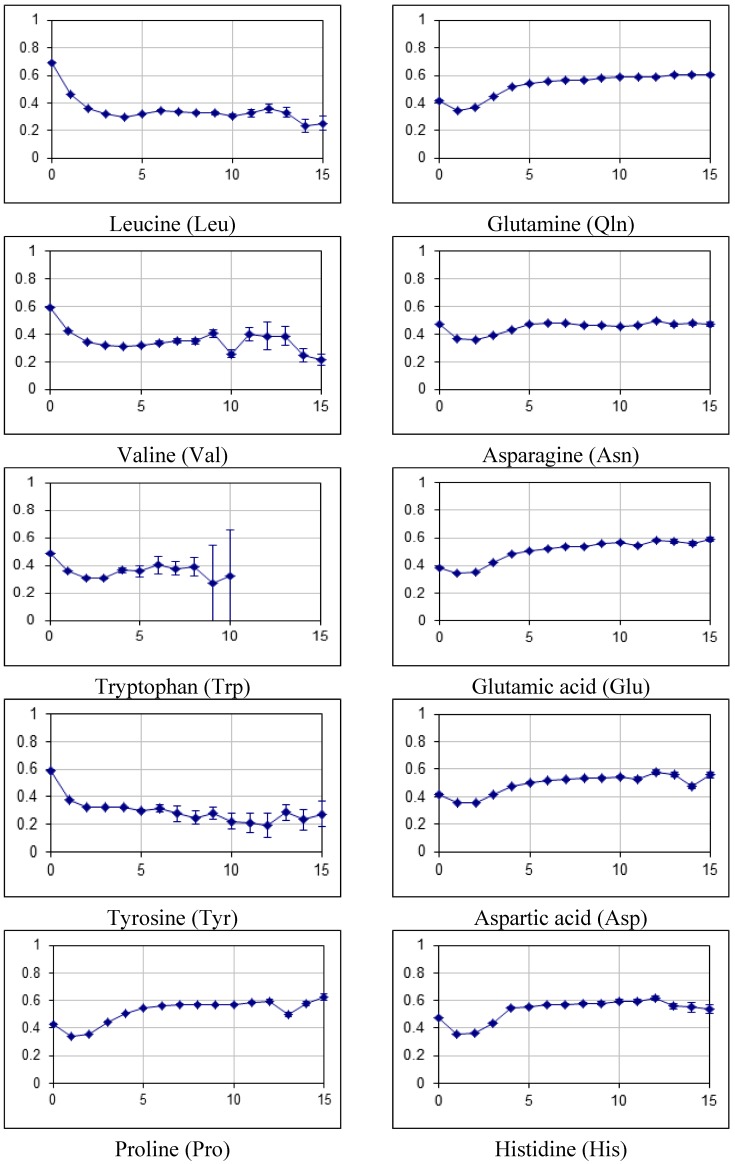

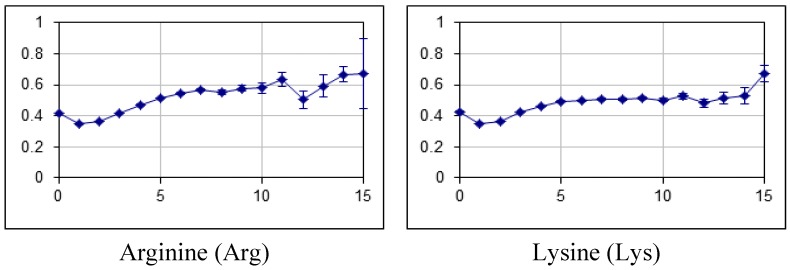
Fraction of disordered residues for proteins containing homo-repeats tabulated for all 20 amino acids. Average fraction of disordered residues *versus* the length of the homo-repeat is plotted for hydrophobic, small, polar and charged amino acids as indicated.

Figure 8 shows fraction of disordered residues *versus* the length of homo-repeats for all 122 proteomes. For hydrophobic amino acids a decreasing fraction of disordered residues, at the same time this value for charge, polar and small amino acid residues was increasing. The maximum fraction of disordered residues was obtained for proteins with lysine and arginine homo-repeats. These values correspond to 0.7. The minimum value corresponds to valine and leucine homo-repeats, these values are about 0.2.

Table 1 shows a big difference between the occurrences of different homo-repeats in the bacterial (*B_japonicum*) and eukaryotic (*H_sapiens*) proteomes. The information about the proteins used in this analysis can be found in the HRaP database. These bacterial proteomes show frequent occurrences of homo-repeats containing small amino acids (alanine, glycine, serine, threonine, and proline), whereas human proteome contains many proteins with homo-repeats containing small, charged and polar amino acids.

**Table 1:** Number of proteins with occurrence of homo-repeats of different lengths (up to 15 residues) for bacterial (*B_japonicum*) and eukaryotic (*H_sapiens*) proteomes.

***B_japonicum***																	
**Amino Acid**	**Homo-Repeat Length**																
	**0**	**1**	**2**	**3**	**4**	**5**	**6**	**7**	**8**	**9**	**10**	**11**	**12**	**13**	**14**	**15**	**>15**
**C**	1453	6768	364	11													
**M**	–	8240	1501	48	4												
**F**	103	8140	2477	119	6												
**I**	22	8229	3507	193	7												
**L**	5	8247	6620	1481	213	23	1										
**V**	9	8239	5807	1010	103	2	–	1									
**W**	930	7312	496	18	1												
**Y**	386	7848	1247	42	2												
**A**	–	8253	7467	3151	586	89	19	2	1								
**G**	8	8242	5904	1202	149	21	10	4	2	–	1	–	–	–	–	–	
**T**	20	8229	4149	432	41	4	2										
**S**	9	8241	4491	525	62	5	2	1	2	–	–	1					
**Q**	107	8138	2265	203	12	–	–	–	–	–	–	–	1				
**N**	195	8043	1563	58	2	–	–	1									
**E**	40	8206	3785	272	10	1	1										
**D**	32	8214	4157	368	24	2	1	2									
**H**	324	7911	1215	87	9	2	1										
**R**	16	8218	5771	968	102	17	3										
**K**	111	8132	2401	157	10	3											
**P**	36	8216	3810	473	76	19	8	5	1	–	–	1					
***B_japonicum***																	
**Amino Acid**	**Homo-Repeat Length**																
	**0**	**1**	**2**	**3**	**4**	**5**	**6**	**7**	**8**	**9**	**10**	**11**	**12**	**13**	**14**	**15**	**>15**
**C**	4183	54,706	10,645	539	82	20	16	1	–	–	1						
**M**	590	58,389	9287	320	17	–	1	2									
**F**	2011	56,921	20,466	1535	146	30	12	10	2	1	1						
**I**	1886	57,053	24,782	2218	118	21	2	1	–	1							
**L**	296	58,704	47,078	15,204	2697	918	185	131	57	39	17	13	–	1	–	1	
**V**	681	58,306	17,587	4911	458	40	9										
**W**	7492	51,379	4293	108	3	1											
**Y**	3959	54,975	14,416	854	53	5	2										
**A**	590	58,370	39,712	10,362	2011	671	311	180	82	81	43	28	18	21	17	9	
**G**	603	58,383	35,065	7075	1460	461	209	138	66	27	30	23	12	7	6	7	
**T**	718	58,220	28,359	3512	416	130	43	14	5	3	–	2	1	1			
**S**	303	58,692	42,712	13,046	2925	699	259	122	69	52	25	24	16	5	10	–	
**Q**	1197	57,773	25,784	3675	567	216	131	63	53	19	46	15	11	4	13	17	
**N**	2414	56,528	18,282	1446	92	21	–	2	–	–	1						
**E**	898	58,037	38,361	10,830	2461	852	381	261	144	86	42	18	17	30	10	14	
**D**	1511	57,465	24,695	1769	405	144	47	17	29	2	–	1	1	2	5	1	
**H**	3230	55,669	13,649	842	116	41	26	26	14	29	9	8	5	2	1	2	
**R**	785	58,172	18,597	6686	1080	212	48	6	3	3	1	1	–	–	–	–	
**K**	1423	57,514	32,501	6424	1083	263	102	47	64	66	18	–	2	3	1	1	
**P**	891	58,076	17,653	8799	2173	774	357	219	114	69	55	26	16	18	2	6	

## 3. Experimental Section

### 3.1. Proteomes

Table 2 lists 122 proteomes used in the current study. These proteomes were also analyzed in our other recent study [16].

**Table 2:** Names of 97 eukaryotic and 25 bacterial proteomes.

Eukaryota		Eukaryota (Fungi)	Bacteria ***	
Metazoa	25.H_sapiens; 22974.B_taurus; 59.M_musculus; 122.R_norvegicus; 21457.G_gallus; 20721.D_rerio; 22388.T_nigroviridis; 17.D_melanogaster; 25396.D_pseudoobscura; 31436.A_aegypti; 78607.A_darlingi; 22426.A_gambiae; 21633.C_briggsae; 9.C_elegans; 64800.L_loa; 79720.T_spiralis; 30565.N_vectensis	34310.A_capsulata_ATCC_26029; 34967.A_capsulata_H143; 34495.A_dermatitidis_SLH14081; 34498.A_dermatitidis_ER-3; 35919.A_benhamiae; 29154.A_clavatus; 33020.A_flavus; 22118.A_fumigatus_FGSC_A1100; 31018.A_fumigatus_CEA10; 29130.A_niger; 23077.A_oryzae; 28239.A_terreus; 30100.B_fuckeliana; 22024.C_albicans_SC5314; 32738.C_dubliniensis; 19665.C_glabrata; 34491.C_tropicalis; 25585.C_globosum_IFO_6347; 34493.C_lusitaniae; 34218.C_posadasii; 79902.C_graminicola; 20018.D_hansenii; 34482.L_thermotolerans; 29447.L_elongisporus; 22028.M_oryzae;	Acidobacteria	25797.S_usitatus 37022.A_mediterranei; 33926.C_acidiphila; 35278.Frankia_sp_EuI1c; 35534.F_sp; 74443.K_setae; 33113.R_opacus; 25456.Rhodococcus_sp; 131.S_avermitilis; 36666.S_bingchenggensis; 84.S_coelicolor; 34910.S_scabies; 58962.S_violaceusniger; 34011.S_roseum
Viridiplantae	23214.O_sativa; 3.A_thaliana; 33157.Micromonas_sp; 29351.O_lucimarinus; 25972.O_tauri	34471.N_otae; 34970.N_haematococca; 29157.N_fischeri; 22025.N_crassa; 34307.P_brasiliensis_Pb03; 34389.P_brasiliensis_Pb18; 34392.P_brasiliensis_ATCC_MYA‑826; 31898.P_chrysogenum;	Proteobacteria	112.B_japonicum; 22343.Burkholderia_sp_ATCC_17760; 25388.B_xenovorans; 33223.H_ochraceum; 23351.M_xanthus; 32044.P_pacifica; 30295.S_cellulosum; 33616.S_aurantiaca
Stramenopiles *	35109.E_siliculosus	32999.P_marneffei 25591.P_nodorum; 29448.P_guilliermondii;	Bacteroidetes	33930.C_pinensis; 32144.M_marina
Choanoflagellida **	30562.M_brevicollis	28727.P_stipitis; 79908.P_graminis;	Chloroflexi	36622.K_racemifer
Amoebozoa *	21395.D_discoideum; 35301.P_pallidum	30091.S_cerevisiae_YJM789; 31651.S_cerevisiae_RM11-1a; 34506.S_cerevisiae_JAY291; 35062.S_cerevisiae_Lalvin_EC1118; 71242.S_cerevisiae; 30103.S_sclerotiorum; 35280.S_macrospora; 33056.T_stipitatus; 35921.T_verrucosum; 34386.U_reesii; 30097.V_polyspora; 35359.V_albo-atrum; 20011.Y_lipolytica; 31020.C_cinerea; 20846.C_neoformans_JEC21; 21380.C_neoformans_B-3501A; 31023.L_bicolor; 33031.P_placenta; 22029.U_maydis		

* Category without rank is given; ** The name of order is given because the highest ranks are missing in the taxonomic description; *** The super-kingdom of bacteria is divided in phyla rather than kingdoms.

### 3.2. Prediction of Disordered Residues

Disordered residues were predicted using the IsUnstruct program, which is based on the Ising model [19]. The parameters of the program were determined and optimized on the basis of protein structural statistics. The tests demonstrated that the program yields reliable predictions [18]. The program is available at our site [18]. It is important that the results of our method (IsUnstruct) are better than the results of the meta-predictor PONDR-FIT [24] and are comparable to the results of the neural-network-based technique CSpritz [25] (see Table 3). Our program works as well as the meta-server programs. This fact is presented in Table 5 from paper [18].

**Table 3:** Performance of disorder prediction methods on DisProt database version 3.7. Predictors are ranked according to AUC.

Method	Sw	AUC
CSpritz [25] (Walsh *et al*., 2011)	0.571	0.877
IsUnstruct [18] version 2.02 (Lobanov *et al*., 2013)	0.567	0.856
IUPred (long) [26] (Dosztanyi *et al.*, 2005)	0.426	0.818
PONDR-FIT [24] (Xue *et al.*, 2010)	0.515	0.817
FoldUnfold [20] (Galzitskaya *et al.*, 2006)	0.446	0.813
DISOPRED [27] (Ward *et al.*, 2004)	0.462	0.806

**Figure 9:**
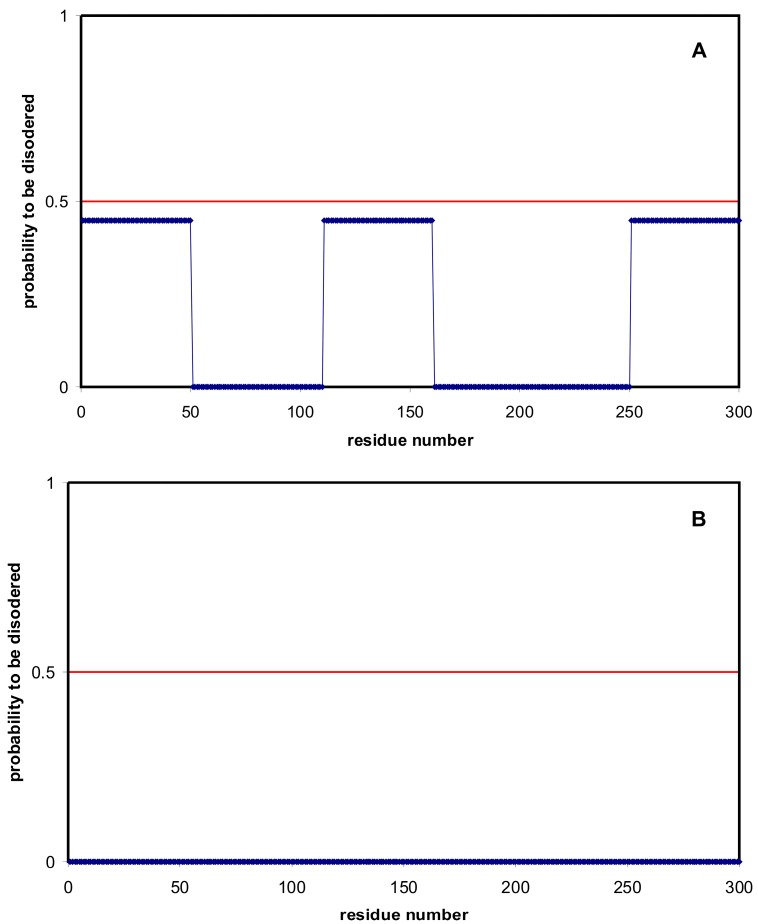
Prediction of disordered residues for artificial proteins (**A**) and (**B**) (blue curves). Red line corresponds the border between disordered and ordered residues, below 0.5 generally indicating ordered residues and above 0.5 intrinsically disordered residues.

We have done additional calculations to demonstrate the definition of the fraction of disordered residues in protein. For artificial protein A there are three predicted disordered regions with the length of 50 residues (Figure 9). The probability to be disordered for each residue from these regions is 0.45. The other residues are predicted as ordered. Protein B is an ordered one by the prediction.

If to use the definition of the fraction of disordered residues as the number of disordered residues divided by the total number of residues [22,28], then both proteins are ordered proteins. If to use our definition of the disordered content as summation of probabilities, then we receive 0.45 × 150/300 = 0.225. What score reflects the reality better? To simplify the situation, let us consider whether the fragments can be entirely disordered or ordered. In addition, we assume that the fragments fold independently. Both assumptions are quite natural for real proteins if the fragments are separated in the space. Then the probability that all of the regions are folded is *p* = (1 − 0.45)^3^ = 0.17. This is the probability that the whole protein is ordered. As you can see, it is very low. The probability that there will be only one fragment disordered is 0.41, [3!/(2! × 1!) × 0.45 × (1 − 0.45)^2^ = 0.408375], 0.33 for 2 fragments [3!/(2! × 1!) × 0.45^2^ × (1 − 0.45) = 0.334125], and all 3 fragments will be disordered with the probability of 0.09 [3!/(3! × 0!) × 0.45^3^ × (1 − 0.45)^0^]. If we make an exhaustive search over all possible states of the protein, calculate the fraction of disordered residues in each state and take into account the probability of states, then we obtain our assessment: the average fraction of disordered residues of protein is 0.225. However, the probability to be completely ordered is very low (0.17 = 3!/(0! × 3!) × 0.45^0^ × (1 − 0.45)^3^).

In the case of real protein, we make an exhaustive search over all possible variants of the partially ordered/disordered proteins using the Ising model. In addition, in this case, the average fraction of disordered residues of protein exactly corresponds to the summation of probabilities normalized by the length of the protein. Or for the proteome, summation of the probabilities for all residues in the database is normalized to the total number of residues in the proteome.

**Figure 10:**
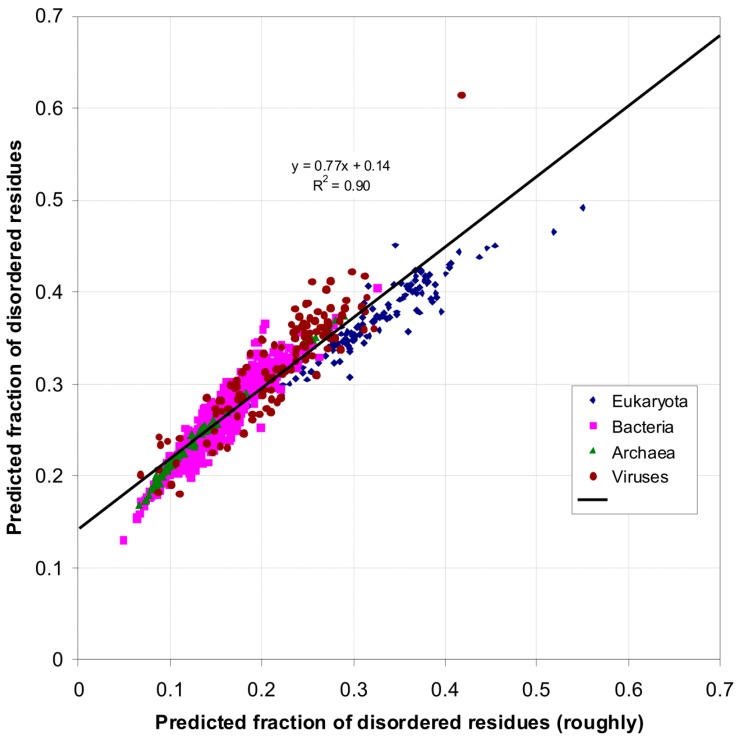
Predicted fraction of disordered residues by using the two definitions.

**Figure 11:**
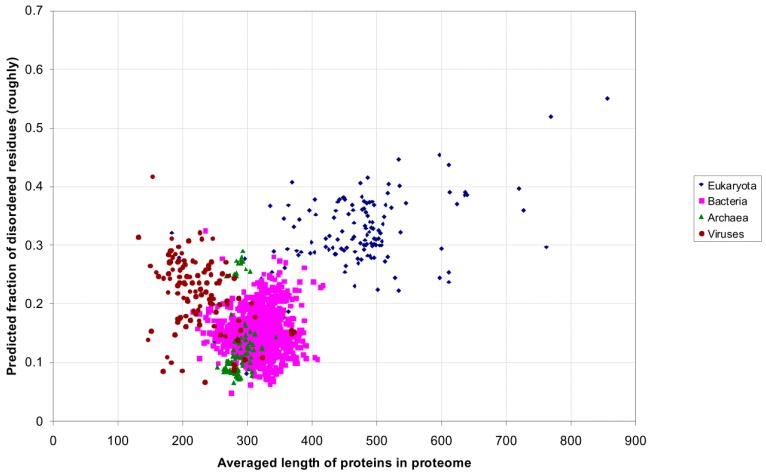
Predicted average fraction of disordered residues for 1902 species from eukaryotes (137), bacteria (1540), archaea (105) and viruses (120) *versus* the average protein length in the proteome using the rough definition.

In real proteins there are many cases when the predicted probability of being disordered is close to 0.5. If we use the rough definition of the fraction of disordered residues (*i.e*., the number of disordered residues divided by the total number of residues), we obtain understated estimates, as one can see from the presented Figure 10 and Figure 11 and Table 4 below.

**Table 4:** Average contents of disorder for 4 domains of life.

Domain	Number	Our Definition	Rough Definition	Difference
Eukaryota	137	0.366	0.319	0.047
Bacteria	1540	0.260	0.151	0.109
Archaea	105	0.236	0.128	0.108
Viruses	120	0.282	0.176	0.106

Figure 10 demonstrates the correlation between the two definitions. If to use the rough definition of the fraction of disordered residues, our result is not changed (see Figure 11). One can see the intersection of the fraction of disordered residues between eukaryotic and bacterial species, only we obtained all understated results.

## 4. Conclusions

The accepted notion in the field is that bacterial proteomes do not contain large numbers of disordered residues. Surprisingly, our analysis of large bacterial proteomes (with the number of residues exceeding 2,500,000) suggests that the fraction of disordered residues is comparable to that in eukaryotic proteomes. A continuous dependence of the fraction of disordered residues on the size of the proteome is observed for four domains of life: Eukaryota, Bacteria, Archaea, and Viruses. Moreover, the fraction of disordered residues increases for proteins with homo-repeats comprised of small, charged, and polar residues, and decreases for hydrophobic residues. We also demonstrate that using the definition of the fraction of disordered residues in proteins as the ratio of disordered residues to the total number of residues, the results are understated as compared to our definition as summation of probabilities for residues to be disordered normalized by the protein length.
